# The ‘cognitive footprint’ of psychiatric and neurological conditions: cross‐sectional study in the UK Biobank cohort

**DOI:** 10.1111/acps.12733

**Published:** 2017-04-07

**Authors:** B. Cullen, D. J. Smith, I. J. Deary, J. J. Evans, J. P. Pell

**Affiliations:** ^1^ Institute of Health and WellbeingUniversity of GlasgowGlasgowUK; ^2^ Centre for Cognitive Ageing and Cognitive EpidemiologyUniversity of EdinburghEdinburghUK

**Keywords:** Cognitive Dysfunction, mood disorders, neurological disorders, prevalence, schizophrenia

## Abstract

**Objective:**

We aimed to quantify the prevalence of cognitive impairment in adults with a history of mood disorder, schizophrenia, multiple sclerosis or Parkinson's disease, within a large general population cohort.

**Method:**

Cross‐sectional study using UK Biobank data (*n *=* *502 642). Psychiatric and neurological exposure status was ascertained via self‐reported diagnoses, hospital records and questionnaires. Impairment on reasoning, reaction time and memory tests was defined with reference to a single unexposed comparison group. Results were standardised for age and gender. Sensitivity analyses examined the influence of comorbidity, education, information sources and missing data.

**Results:**

Relative to the unexposed group, cognitive impairment was least common in major depression (standardised prevalence ratios across tests = 1.00 [95% CI 0.98, 1.02] to 1.49 [95% CI 1.24, 1.79]) and most common in schizophrenia (1.89 [95% CI 1.47, 2.42] to 3.92 [95% CI 2.34, 6.57]). Prevalence in mania/bipolar was similar to that in multiple sclerosis and Parkinson's disease. Estimated population attributable prevalence of cognitive impairment was higher for major depression (256 per 100 000 [95% CI 130, 381]) than for all other disorders.

**Conclusion:**

Although the relative prevalence of cognitive impairment was lowest in major depression, the population attributable prevalence was highest overall for this group.

**Significant outcomes**

- Cognitive impairment in bipolar disorder has previously received less research and clinical attention than impairment in schizophrenia and neurological disorders, but direct comparisons in the present study indicated that impairment prevalence in mania/bipolar disorder was similar to that in multiple sclerosis and Parkinson's disease, both of which are much less common in the population.
- As in previous clinical studies, cognitive impairment was found to be most common in participants with schizophrenia and least common in those with major depression.
- The high population prevalence of major depression means that the overall burden of cognitive impairment attributable to this disorder is likely to be considerable.

**Limitations**

- Information regarding exposure status relied substantially on self‐reported diagnoses or responses to questionnaire items.
- The cognitive tests were brief and did not assess long‐term episodic memory or complex executive skills, which are often impaired in people with psychiatric or neurological conditions.
- The UK Biobank cohort is not representative of the UK population in some respects, and the exposure groups that we identified within it are likely to differ from psychiatric and neurological samples in other studies and in clinical practice, with regard to sociodemographic characteristics, illness severity and motivational factors.

## Introduction

Psychiatric and neurological conditions are associated with cognitive impairment, which contributes to chronic disability, reduced wellbeing and quality of life, and restricted social and economic participation. This ‘cognitive footprint’ 1 will be evident over many years, with impact being particularly marked in common conditions and in those with younger age at onset. Cognitive impairment has long been a focus of research in chronic neurological conditions such as multiple sclerosis (MS) and Parkinson's disease (PD), as well as severe psychiatric disorders such as schizophrenia, with attention turning more recently to the cognitive impact of common mental health conditions. The cognitive burden of these chronic conditions is of great public health importance: interventions to prevent or manage associated cognitive impairment have the potential ‘to foster cognitive health and to preserve cognitive capital’ 1 at both individual and societal levels. Mood disorders, schizophrenia, MS and PD are particularly important to consider in this context: unlike the dementias, average age of onset of these disorders is under 65 years, and their chronic illness course means that individuals may live with cognitive impairment for many years.

In people with mood disorders, for example, cognitive impairment persists between illness episodes and contributes substantially to ongoing disability 2, 3. This impacts negatively on functional independence, educational and occupational attainment, and quality of life 4, 5, 6, 7, and cognitive improvement is therefore an increasing focus of treatment development efforts 8, 9, 10. Estimates of cognitive impairment prevalence in euthymic adults with mood disorder vary considerably, ranging from 5% to 58% in adults with bipolar disorder (BD) 11, and from one‐third to one‐half in those with major depressive disorder 12. Cognitive impairment is evident in the majority of people with schizophrenia – possibly as many as 80% – and has been proposed as a core diagnostic criterion 13, 14. Community‐based studies have reported cognitive impairment prevalence of 44%–48% in people with MS 15 and 50%–55% in those with PD 16.

Comparative burden of cognitive impairment across disorders must be considered with reference to the relative prevalence of the disorders themselves. In the United Kingdom, lifetime prevalence per 100 000 population is estimated at up to 10 000 for major depression 17 and approximately 1 000 for BD 18, compared with 400–1 000 for schizophrenia 19 and 200 for both MS and PD 20. Despite the greater research focus on cognitive function in schizophrenia and neurological disorders, it is clear that the higher prevalence of mood disorders in the population means that the absolute number with associated cognitive impairment is likely to be substantial and of significant public health importance.

Previous studies of cognitive function in various psychiatric and neurological conditions are difficult to compare directly, because of variations in source populations, methods of recruitment, assessment tools, impairment definitions, composition of normative comparison groups and adjustment for potential confounders. The UK Biobank general population cohort presents an opportunity to overcome these limitations. UK Biobank recruited more than half a million adults in middle and early old‐age, with baseline assessment of medical history, cognitive function, sociodemographic characteristics and lifestyle factors, as well as linkage to hospital records 21. It is therefore possible to compare cognitive impairment prevalence directly across conditions, using the same measures and impairment thresholds, taking account of confounders that have been measured in a standardised way.

### Aims of the study

In this cross‐sectional study, we aimed to quantify the prevalence of cognitive impairment in adults with a history of mood disorder, schizophrenia, MS or PD, in the UK Biobank cohort. Standardised direct comparisons will contribute to a clearer picture of the relative frequency of cognitive impairment in these conditions, as well as the magnitude of attributable prevalence at the population level. This enables both a disorder‐specific and a general population‐level appreciation of the ‘cognitive footprint’ of key psychiatric and neurological conditions affecting working‐age adults.

## Material and methods

### Participants

Adults aged 40–69 years who were registered with the National Health Service (NHS) and living within 25 miles of a study assessment centre were invited by post to participate in UK Biobank, with a response rate of approximately 6% 22. Data from the full cohort at baseline (*n *=* *502 642) were used in the present study. All participants gave written informed consent. This study was conducted under generic approval from the NHS National Research Ethics Service (Ref. 11/NW/0382), and the data were obtained from UK Biobank under application 11332.

### Materials and procedure

Baseline assessments took place at 22 centres across England, Scotland and Wales between 2006 and 2010. Participants attended a single visit lasting approximately 2–3 h, encompassing consent processes, computerised touch screen questionnaire, nurse interview and physical measurements. Assessments were administered in a standardised order. Key sociodemographic information for the present study included age, gender and educational attainment (dichotomised according to whether or not participants held a university/college degree). Full details of demographic, lifestyle and psychological measures are given in Appendix S4 and Appendix S1. Information regarding minimisation of bias in the study procedures is provided in Appendix S4.

#### History of psychiatric and neurological conditions

Psychiatric and neurological conditions were studied as risk factors for the outcome of cognitive impairment. The conditions of interest in the present study were mania/BD, major depression (single or recurrent episodes), schizophrenia, MS and PD. Three data sources were used to ascertain whether participants were ‘exposed’ to any of the conditions of interest: self‐reported diagnoses; linked NHS hospital records; and a touch screen mood disorders questionnaire.

##### Self‐reported diagnoses

All participants were asked whether they had ever been told by a doctor that they had a serious illness or disability. Responses were recorded during the nurse interview and were subsequently assigned unique codes. Conditions of interest were manually coded for the present study, as listed in Appendix S2. Participants reporting both mania/BD (‘mania/bipolar/manic depression’) and major depression (‘depression’ or ‘postnatal depression’) were classified as mania/BD.

##### Linked hospital records

Medical history was also available from hospital records, retrieved by UK Biobank from NHS data providers across the UK and linked centrally to UK Biobank data using participant identifiers. Data were analysed from in‐patient and day‐case admissions to NHS hospitals between the mid‐1990s (varied by country) and the UK Biobank baseline assessment date. ICD‐10 codes from primary or secondary positions in the hospital records were used to identify participants with conditions of interest (Appendix S3). Hospital records from England and Wales included both general and psychiatric hospitals, but records from Scotland covered general hospitals only. Participants in Scotland (7.1% of the whole cohort) were therefore coded as ‘missing’ when ascertaining the mania/BD, major depression and schizophrenia groups using ICD data, but they contributed ICD data when ascertaining the MS and PD groups. Participants with ICD‐10 codes for both mania/BD (F30x or F31x) and major depression (F32x or F33x) were classified as mania/BD.

##### Mood disorders questionnaire

A third ascertainment method was available to identify history of mania/BD or major depression, based on the UK Biobank baseline touch screen questionnaire (final 2 years of baseline recruitment only). As described by our group previously 23, responses to questions regarding lifetime experience of depressive and manic symptoms and medical help‐seeking for mental health were used to identify participants with a probable lifetime history of mania/BD or major depression. Participants meeting criteria for both disorders were classified as mania/BD. We have previously described cognitive performance in these groups 24.

##### Exposed and unexposed group definitions

Exposure status for each condition of interest (mania/BD, major depression, schizophrenia, MS and PD) was classified in two ways, using ‘broad’ and ‘narrow’ definitions. Participants who met criteria for the condition according to at least one ascertainment source were classified in the broadly defined exposure group, and participants who met criteria according to at least two ascertainment sources were classified in the narrowly defined exposure group.

A single comparison group was constructed as a common reference for all of the exposure groups. Participants in this group were considered to be unexposed with regard to the conditions of interest in this study, as well as any other psychiatric condition or any condition affecting brain function. This group met all of the following criteria: provided data on the mood disorders questionnaire and were not classified in the mania/BD or major depression groups (mild subthreshold symptoms were permitted); no hospital ICD‐10 code of any psychiatric or brain condition (as listed in Appendix S3); no self‐reported diagnosis of any psychiatric or brain condition (as listed in Appendix S2). Participants who did not meet criteria for any of the exposed or unexposed groups were not further analysed.

#### Cognitive function

Cognitive tests were administered visually via a touch screen. The tests are described briefly below, and full details are provided in Appendix S4.

##### Reasoning

Verbal and numerical problems were presented on‐screen, and participants were asked to choose the correct response from multiple options. Although referred to as the ‘fluid intelligence’ test in the UK Biobank protocol, performance on some of the items was thought to rely on crystallised knowledge, and so we refer to it simply as a reasoning test. The score was an unweighted total of correct responses, from 0 to 13.

##### Reaction time

Psychomotor speed was measured by pressing a button as quickly as possible each time a matching pair of symbols was presented on‐screen. The score was the mean time in milliseconds across trials.

##### Numeric memory

This test was intended to assess working memory. A string of numbers was presented on‐screen, and after a brief delay, participants were asked to enter it from memory, in reverse order, via a numeric keypad. The score was the maximum string length recalled correctly.

##### Pairs matching

In this test of visual memory, symbol cards were presented on‐screen in a random array. Participants were asked to memorise the position of matching pairs. The cards were then turned face down, and participants were asked to touch as many matching pairs as possible in the fewest tries. The score was the number of errors made whilst attempting to select the pairs.

##### Prospective memory

An instruction was given on‐screen asking participants to select a certain shape from an array that would be presented later in the assessment process. When the array later appeared, participants were given up to two chances to select the correct shape. Performance was scored dichotomously as being correct on the first attempt or not.

Participants in the exposed groups were classified as impaired if their score was equal to or worse than the lowest‐performing 5% (or nearest feasible proportion) of the unexposed group (see Appendix S4). Since prospective memory was a categorical measure, impairment in all groups was defined as being incorrect on the first attempt.

### Data analysis

Prevalence of impairment on the five cognitive tests was calculated in each group, reported as a percentage with 95% confidence intervals (CI) based on the standard error calculated as follows: √((prevalence*(100‐prevalence))/*n*). The ratio of prevalence in each exposed group vs. the unexposed reference group was calculated, together with 95% CI, using the epitab functions in Stata v13. Crude results are reported, along with directly standardised results, which were computed using weights derived from the unexposed comparison group. In direct standardisation, sample sizes within each stratum of an external reference group (here, the unexposed comparison group) are used as weights to adjust the stratum‐specific prevalence ratios in the exposed group: the prevalence ratio in each stratum of the exposed group is multiplied by the sample size of that stratum in the reference group, and the resulting products are then summed and divided by the total sample size of the reference group, to produce a standardised prevalence ratio for the whole exposed group. The purpose of standardisation was to control for demographic differences between the exposed and unexposed groups that might confound the crude results; stratification was by age group (<60 vs. ≥60 years) and gender. Statistical interactions between exposure status and each of the confounders were tested using robust Poisson regression models 25 including a product term. Other descriptive analyses are detailed in Appendix S4.

The population attributable prevalence of cognitive impairment (number of cases of cognitive impairment per 100 000 total population that are attributable to the exposure) was derived from 2 × 2 tables constructed for each exposure separately. Prevalence of each exposure within these tables was based on lifetime estimates cited in the Introduction. Population attributable prevalence was calculated as total cases of cognitive impairment per 100 000 population * population attributable fraction (PAF), where PAF was (prevalence_total_‐prevalence_unexposed_)/prevalence_total_.

Sensitivity analyses were conducted to examine the effects of several potential sources of bias and confounding, including comorbidity, education, information sources and missing data. Details are provided in Appendix S4. Reporting follows STROBE guidance 26.

## Results

### Characteristics of the exposed and unexposed groups

Table 1 shows the characteristics of each group on key demographic factors and the five cognitive measures. Additional group characteristics are provided in Table S1. The total number in the cohort who did not meet criteria for any of the exposed groups or the unexposed comparison group was 336 662; a large number were excluded from the unexposed group because they were not administered the mood disorders questionnaire. The relative sizes of the broadly and narrowly defined versions of each exposure group were closer for MS and PD than for the other exposures, possibly reflecting the greater likelihood of a hospital admission during the available time period for participants with these diagnoses. The proportion of missing data varied across measures, but tended to be higher in the exposed groups (particularly those with schizophrenia), compared with the unexposed comparison group. Cronbach's alpha is reported in Table 1 for the reasoning and reaction time tests in each group; it was not possible to calculate this for the other three cognitive tests, as they did not include multiple items. Alpha was higher for reaction time than for reasoning, but coefficients did not differ notably across groups. Other psychometric characteristics of the cognitive tests have been reported by us previously 27.

**Table 1:** Characteristics of the exposed and unexposed groups

	Unexposed comparison	Mania/bipolar		Major depression		Schizophrenia		Multiple sclerosis		Parkinson's disease	
		Broad	Narrow	Broad	Narrow	Broad	Narrow	Broad	Narrow	Broad	Narrow
*n*	104 410	3020	607	56 425	7583	850	259	1905	931	916	323
Age (years)^a^											
Mean (SD)	57.0 (8.2)	54.9 (8.0)	54.8 (8.0)	55.6 (7.9)	55.1 (7.9)	53.9 (8.1)	52.6 (8.2)	55.4 (7.5)	55.3 (7.5)	62.2 (5.6)	62.5 (5.2)
Gender^a^											
*n* (%) female	52 210 (50.0)	1589 (52.6)	345 (56.8)	36 574 (64.8)	4923 (64.9)	293 (34.5)	74 (28.6)	1400 (73.5)	665 (71.4)	346 (37.8)	131 (40.6)
Has a degree											
*n* (%) missing	1033 (1.0)	40 (1.3)	4 (0.7)	582 (1.0)	59 (0.8)	31 (3.7)	10 (3.9)	39 (2.0)	26 (2.8)	20 (2.2)	6 (1.9)
*n* (%)^b^	36 082 (34.9)	1117 (37.5)	250 (41.5)	18 188 (32.6)	2344 (31.2)	212 (25.9)	65 (25.3)	642 (34.4)	278 (30.7)	276 (30.8)	88 (27.8)
No known comorbidity^a^^,^ c											
*n* (%)		2018 (66.8)	338 (55.7)	43 289 (76.7)	4842 (63.9)	30 (3.5)	8 (3.1)	279 (14.7)	116 (12.5)	172 (18.8)	65 (20.1)
Reasoning score											
*n* (%) missing^d^	1595 (1.6)	40 (2.1)	7 (2.2)	485 (1.4)	97 (1.6)	17 (5.8)	5 (5.5)	17 (2.9)	10 (3.6)	6 (2.1)	2 (1.7)
Mean (SD)	6.0 (2.2)	5.7 (2.2)	6.0 (2.4)	6.0 (2.1)	5.9 (2.2)	4.8 (2.1)	4.7 (2.0)	5.9 (2.0)	5.7 (2.0)	5.8 (2.2)	5.7 (2.2)
Cronbach's α	0.70	0.71	0.73	0.69	0.69	0.70	0.65	0.66	0.64	0.70	0.71
Reaction time (ms)											
*n* (%) missing	1107 (1.1)	65 (2.2)	14 (2.3)	652 (1.2)	99 (1.3)	52 (6.1)	16 (6.2)	39 (2.1)	25 (2.7)	19 (2.1)	7 (2.2)
Median (Q1, Q3)	543 (484, 621)	558 (492, 640)	571 (500, 664)	543 (484, 621)	551 (488, 633)	601 (520, 688)	583 (521, 668)	590 (512, 694)	594 (516, 716)	571 (512, 644)	578 (516, 653)
Cronbach's α	0.82	0.83	0.85	0.82	0.83	0.87	0.85	0.84	0.85	0.82	0.83
Numeric memory score											
*n* (%) missing^d^	749 (2.4)	22 (4.2)	2 (2.6)	301 (2.7)	59 (3.3)	9 (12.5)	5 (20.0)	9 (4.5)	8 (7.2)	3 (3.1)	2 (5.4)
Mean (SD)	6.7 (1.3)	6.5 (1.5)	6.5 (1.3)	6.7 (1.3)	6.6 (1.4)	5.9 (1.5)	5.9 (1.3)	6.6 (1.3)	6.4 (1.3)	6.5 (1.4)	6.4 (1.5)
Pairs matching errors											
*n* (%) missing	0 (0.0)	14 (0.5)	1 (0.2)	117 (0.2)	12 (0.2)	17 (2.0)	3 (1.2)	18 (0.9)	11 (1.2)	9 (1.0)	2 (0.6)
Median (Q1, Q3)	3 (2, 5)	4 (2, 6)	4 (2, 7)	3 (2, 6)	4 (2, 6)	4 (2, 7)	4 (2, 6)	3 (2, 6)	3 (2, 6)	4 (2, 6)	4 (2, 7)
Prospective memory^a^^,^ d											
*n* (%)^a^ correct	80 192 (77.2)	1391 (71.0)	219 (65.4)	28 148 (77.7)	4724 (75.4)	180 (55.4)	58 (56.3)	446 (73.4)	206 (70.8)	217 (70.9)	79 (63.7)

Q, quartile; SD, standard deviation.

a No missing data.

b Missing excluded from denominator.

c Not known to have any other psychiatric or brain condition in addition to the exposure. By definition, no member of the unexposed comparison group had any primary or comorbid psychiatric or brain condition.

d Missing data refer only to the period when this measure was included in the battery.

### Prevalence of cognitive impairment across groups

On the reaction time, numeric memory and pairs matching tests, the cognitive impairment threshold corresponded to the worst‐performing 5% of the unexposed group. Owing to the restricted raw score range on the reasoning test, the same raw score spanned the 5th to 11th percentile range and so the 4th percentile score was instead used to divide the sample; the impairment threshold therefore corresponded to the worst‐performing 4% of the unexposed group. Prospective memory was a pass/fail test; the proportion of the unexposed group with an incorrect score was 22.82%. Table 2 shows prevalence of impairment in each exposed group, along with prevalence ratios relative to the unexposed group. Standardised estimates are not reported for some groups, because of insufficient data in some strata.

**Table 2:** Prevalence of cognitive impairment across groups

		Mania/bipolar		Major depression		Schizophrenia		Multiple sclerosis		Parkinson's disease	
Impairment threshold		Broad	Narrow	Broad	Narrow	Broad	Narrow	Broad	Narrow	Broad	Narrow
**Reasoning**	*n*	1866	318	35 211	6052	274	86	572	272	283	115
≤unexposed 4th percentile score (Unexposed prevalence 4.16%)	Crude P % 95% CI Crude PR 95% CI	7.13 5.96, 8.30 1.71^a^ 1.45, 2.02	6.29 3.62, 8.96 1.51 0.99, 2.31	4.22 4.01, 4.43 1.02 0.96, 1.08	5.24 4.68, 5.80 1.26^a^ 1.13, 1.41	10.95 7.25, 14.65 2.63^a^ 1.88, 3.70	6.98 1.59, 12.37 1.68 0.78, 3.63	2.80 1.45, 4.15 0.67 0.41, 1.09	1.84 0.24, 3.44 0.44 0.19, 1.05	4.95 2.42, 7.48 1.19 0.71, 1.98	6.96 2.31, 11.61 1.67 0.86, 3.27
	Standardised P % 95% CI Standardised PR 95% CI	7.20 6.02, 8.37 1.73^a^ 1.45, 2.05	6.49 3.78, 9.20 1.56^a^ 1.01, 2.42	4.33 4.11, 4.54 1.04^b^ 0.98, 1.10	5.41 4.84, 5.98 1.30^a^ 1.16, 1.47	10.61 6.96, 14.25 2.55^a^ 1.75, 3.71	c	3.20 1.76, 4.65 0.77 0.43, 1.37	c	5.91 3.16, 8.65 1.42 0.70, 2.89	9.24 3.94, 14.53 2.22^a^ 1.02, 4.83
**Reaction time**	*n*	2955	593	55 773	7484	798	243	1866	906	897	316
>unexposed 95th percentile score (Unexposed prevalence 4.98%)	Crude P % 95% CI Crude PR 95% CI	6.73 5.83, 7.63 1.35^a^ 1.18, 1.55	7.59 5.46, 9.72 1.52^a^ 1.15, 2.02	5.31 5.12, 5.50 1.07^a^ 1.02, 1.11	6.55 5.99, 7.11 1.32^a^ 1.20, 1.44	12.91 10.58, 15.24 2.59^a^ 2.16, 3.11	10.70 6.81, 14.59 2.15^a^ 1.49, 3.09	14.31 12.72, 15.90 2.87^a^ 2.56, 3.22	17.77 15.28, 20.26 3.57^a^ 3.10, 4.12	6.69 5.05, 8.33 1.34^a^ 1.05, 1.72	7.28 4.42, 10.14 1.46 0.99, 2.17
	Standardised P % 95% CI Standardised PR 95% CI	7.27 6.33, 8.21 1.46^a^ 1.27, 1.68	8.12 5.92, 10.32 1.63^a^ 1.22, 2.19	5.58 5.39, 5.77 1.12^a^^,^ d 1.07, 1.17	7.02 6.44, 7.60 1.41^a^^,^ e 1.29, 1.55	13.70 11.31, 16.08 2.75^a^ 2.25, 3.37	12.10 8.00, 16.20 2.43^a^ 1.60, 3.69	15.19 13.56, 16.82 3.05^a^^,^ f 2.68, 3.48	18.82 16.28, 21.37 3.78^a^^,^ g 3.22, 4.43	6.27 4.69, 7.86 1.26 0.92, 1.71	5.68 3.13, 8.23 1.14 0.71, 1.83
**Numeric memory**	*n*	505	75	10 808	1746	63	20	193	103	94	35
≤unexposed 5th percentile score (Unexposed prevalence 5.22%)	Crude P % 95% CI Crude PR 95% CI	10.30 7.65, 12.95 1.97^a^ 1.52, 2.56	5.33 0.25, 10.41 1.02 0.39, 2.65	5.65 5.21, 6.09 1.08 0.99, 1.19	7.67 6.42, 8.92 1.47^a^ 1.24, 1.74	20.63 10.64, 30.62 3.95^a^ 2.43, 6.43	15.00 0.00, 30.65 2.87^a^ 1.01, 8.17	6.74 3.20, 10.28 1.29 0.76, 2.19	8.74 3.29, 14.19 1.67 0.90, 3.13	8.51 2.87, 14.15 1.63 0.84, 3.17	14.29 2.70, 25.88 2.74^a^ 1.21, 6.17
	Standardised P % 95% CI Standardised PR 95% CI	10.39 7.73, 13.05 1.99^a^ 1.51, 2.62	c	5.74 5.30, 6.18 1.10 0.99, 1.21	7.78 6.52, 9.03 1.49^a^ 1.24, 1.79	20.46 10.50, 30.42 3.92^a^ 2.34, 6.57	c	7.41 3.72, 11.11 1.42 0.72, 2.82	c	12.68 5.96, 19.41 2.43^a^^,^ h 1.19, 4.97	18.69 5.77, 31.60 3.58^a^^,^ i 1.60, 8.03
**Pairs matching**	*n*	3006	606	56 308	7571	833	256	1887	920	907	321
>unexposed 95th percentile score (Unexposed prevalence 4.38%)	Crude P % 95% CI Crude PR 95% CI	6.35 5.48, 7.22 1.45^a^ 1.26, 1.67	8.58 6.35, 10.81 1.96^a^ 1.51, 2.55	4.76 4.58, 4.94 1.09^a^ 1.04, 1.14	5.15 4.65, 5.65 1.18^a^ 1.06, 1.30	9.12 7.16, 11.08 2.08^a^ 1.68, 2.59	7.81 4.52, 11.10 1.78^a^ 1.17, 2.72	6.20 5.11, 7.29 1.42^a^ 1.19, 1.69	7.28 5.60, 8.96 1.66^a^ 1.32, 2.10	8.27 6.48, 10.06 1.89^a^ 1.52, 2.35	8.10 5.12, 11.08 1.85^a^ 1.28, 2.68
	Standardised P % 95% CI Standardised PR 95% CI	6.57 5.68, 7.46 1.50^a^ 1.30, 1.73	8.98 6.70, 11.26 2.05^a^ 1.57, 2.69	5.12 4.94, 5.31 1.17^a^ 1.12, 1.23	5.65 5.13, 6.17 1.29^a^ 1.16, 1.44	9.68 7.67, 11.69 2.21^a^ 1.75, 2.79	8.58 5.15, 12.02 1.96^a^ 1.20, 3.18	6.57 5.45, 7.69 1.50^a^ 1.22, 1.84	8.02 6.26, 9.77 1.83^a^ 1.41, 2.37	6.66 5.04, 8.28 1.52^a^ 1.16, 1.99	7.36 4.50, 10.21 1.68^a^ 1.05, 2.68
**Prospective memory**	*n*	1959	335	36 237	6267	325	103	608	291	306	124
Incorrect score (Unexposed prevalence 22.82%)	Crude P % 95% CI Crude PR 95% CI	28.99 26.98, 31.00 1.27^a^ 1.18, 1.36	34.63 29.53, 39.73 1.52^a^ 1.31, 1.76	22.32 21.89, 22.75 0.98 0.96, 1.00	24.62 23.55, 25.69 1.08^a^ 1.03, 1.13	44.62 39.22, 50.02 1.95^a^ 1.73, 2.21	43.69 34.11, 53.27 1.91^a^ 1.54, 2.38	26.64 23.13, 30.15 1.17^a^ 1.02, 1.33	29.21 23.99, 34.43 1.28^a^ 1.07, 1.53	29.08 23.99, 34.17 1.27^a^ 1.07, 1.52	36.29 27.83, 44.75 1.59^a^ 1.26, 2.01
	Standardised P % 95% CI Standardised PR 95% CI	29.67 27.64, 31.69 1.30^a^^,^ j 1.20, 1.39	35.37 30.25, 40.49 1.55^a^ 1.34, 1.80	22.82 22.39, 23.25 1.00^k^ 0.98, 1.02	25.56 24.48, 26.64 1.12^a^^,^ l 1.07, 1.17	45.87 40.45, 51.29 2.01^a^ 1.76, 2.28	43.13 33.57, 52.69 1.89^a^ 1.47, 2.42	26.24 22.75, 29.74 1.15 0.99, 1.34	29.67 24.42, 34.91 1.30^a^ 1.07, 1.59	27.84 22.82, 32.86 1.22 0.98, 1.54	34.46 26.09, 42.82 1.51^a^ 1.13, 2.01

CI, confidence interval; P, prevalence; PR, prevalence ratio.

Standardised estimates are directly standardised by age and gender with reference to the unexposed comparison group.

a Significant at *P *<* *0.05 (two‐tailed).

b Significant interaction with gender: women PR = 0.95 (CI 0.88, 1.02); men PR = 1.14 (CI 1.04, 1.25).

c Estimates not reported because at least 1 of 4 strata contained no exposed participants with impairment.

d Significant interaction with gender: women PR = 1.05 (CI 0.99, 1.11); men PR = 1.21 (CI 1.13, 1.30).

e Significant interaction with gender: women PR = 1.29 (CI 1.15, 1.45); men PR = 1.56 (CI 1.34, 1.82).

f Significant interaction with age and gender: <60 years PR = 4.31 (CI 3.65, 5.08); ≥60 years PR = 2.38 (CI 1.94, 2.90); women PR = 2.55 (CI 2.21, 2.93); men PR = 3.69 (CI 2.99, 4.55).

g Significant interaction with age and gender: <60 years PR = 5.85 (CI 4.84, 7.08); ≥60 years PR = 2.66 (CI 2.04, 3.47); women PR = 3.00 (CI 2.50, 3.60); men PR = 4.76 (CI 3.72, 6.08).

h Significant interaction with age: <60 years PR = 4.85 (CI 2.11, 11.17); ≥60 years PR = 0.72 (CI 0.24, 2.19).

i Significant interaction with age: <60 years PR = 6.66 (CI 2.54, 17.50); ≥60 years PR = 1.40 (CI 0.36, 5.51).

j Significant interaction with age: <60 years PR = 1.43 (CI 1.31, 1.56); ≥60 years PR = 1.19 (CI 1.06, 1.33).

k Significant interaction with gender: women PR = 0.96 (CI 0.93, 0.99); men PR = 1.04 (CI 1.00, 1.08).

l Significant interaction with gender: women PR = 1.06 (CI 0.99, 1.12); men PR = 1.19 (CI 1.10, 1.28).

The crude estimates indicated that impairment prevalence was higher in mania/BD than in the comparison group, with prevalence ratios in the broadly defined group ranging from 1.27 (95% CI 1.18, 1.36) to 1.97 (95% CI 1.52, 2.56). Prevalence in major depression was closer to the comparison group level, with crude ratios in the broadly defined group ranging from 0.98 (95% CI 0.96, 1.00) to 1.09 (95% CI 1.04, 1.14). Crude impairment prevalence was higher in MS and PD groups than in the comparison group on reaction time (2.87 [95% CI 2.56, 3.22] and 1.34 [95% CI 1.05, 1.72] respectively in the broadly defined groups), pairs matching (1.42 [95% CI 1.19, 1.69] and 1.89 [95% CI 1.52, 2.35]) and prospective memory (1.17 [95% CI 1.02, 1.33] and 1.27 [95% CI 1.07, 1.52]). Crude impairment prevalence was highest in schizophrenia on all tests (prevalence ratios in the broadly defined group ranged from 1.95 [95% CI 1.73, 2.21] to 3.95 [95% CI 2.43, 6.43]) except reaction time, for which prevalence was highest in MS.

**Figure 1:**
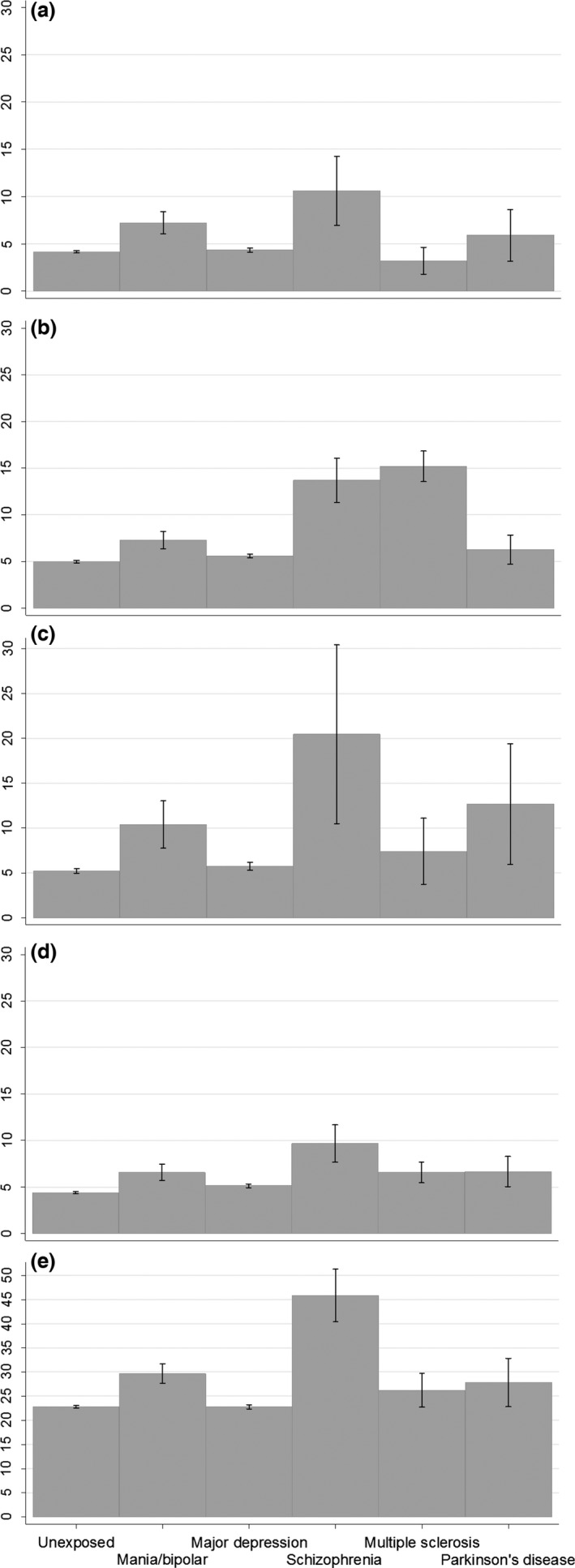
Standardised prevalence estimates for cognitive impairment. Estimates are prevalence (%), directly standardised by age and gender with reference to the unexposed comparison group. Point estimates and 95% confidence intervals are shown. Exposure groups are broadly defined (classified as exposed by at least one ascertainment method). Panels show (a) reasoning; (b) reaction time; (c) numeric memory; (d) pairs matching; (e) prospective memory.

**Figure 2:**
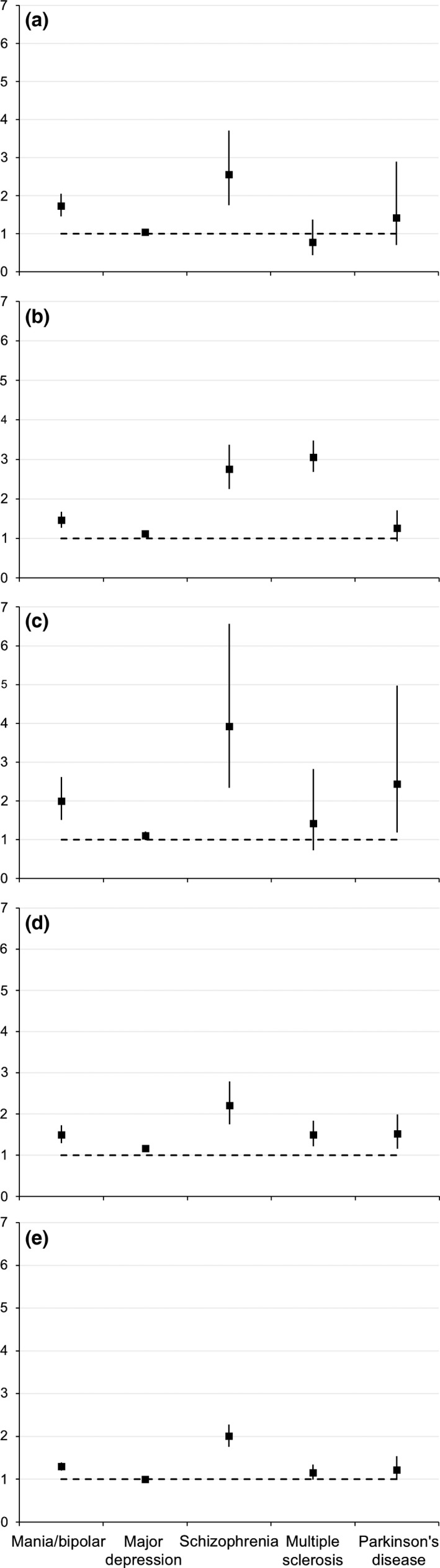
Standardised prevalence ratios for cognitive impairment. Estimates are prevalence ratios compared to the unexposed group, directly standardised by age and gender. Point estimates and 95% confidence intervals are shown. Exposure groups are broadly defined (classified as exposed by at least one ascertainment method). Panels show (a) reasoning; (b) reaction time; (c) numeric memory; (d) pairs matching; (e) prospective memory.

Following direct standardisation with reference to the unexposed group weights for age group and gender, estimates increased in the mania/BD and major depression groups on all measures. Some estimates increased and some decreased in the schizophrenia groups, although the general pattern was similar to the crude results. In MS groups, all estimates but one increased, and in PD, reasoning and numeric memory estimates increased but other estimates decreased. Figure 1 shows the standardised prevalence, and Fig. 2 shows the standardised prevalence ratios on all measures in the broadly defined exposure groups.

Evidence of effect measure modification by age group or gender is highlighted in Table 2, and stratified results are provided in the footnote. Within mania/BD, MS and PD groups, interaction tests indicated that impairment prevalence was significantly lower in the older age group on some measures. Significant interactions with gender were found in major depression and MS on some measures, showing lower impairment prevalence in women.

When the highest standardised impairment prevalence estimates from Table 2 were applied to population prevalence estimates for each illness exposure (cited in the Introduction), the population attributable lifetime prevalence of cognitive impairment per 100 000 population was approximately 256 (95% CI 130, 381) for major depression, 151 (95% CI 52, 251) for schizophrenia, 45 (95% CI 23, 68) for mania/BD, 27 (95% CI 22, 32) for MS and 26 (95% CI 1, 52) for PD.

### Sensitivity analyses

Details of all sensitivity analysis results are provided in Appendix S5. The key findings were that crude estimates in the major depression group attenuated when results were re‐calculated in participants with no known comorbidities; standardised results remained similar when education was taken into account along with age group and gender (Table S2); estimates were higher in alternative mania/BD and major depression groups formed without reference to the mood disorders questionnaire (Table S3); the characteristics of the MS and PD groups identified via hospital records were very similar regardless of whether Scotland data were included; and participants with missing cognitive data were older, less likely to have a degree, and more likely to have comorbidities.

## Discussion

In this community‐based population in middle to early old‐age, standardised prevalence of cognitive impairment was higher in people with a history of psychiatric or neurological conditions than in those with no such history. Across the exposure groups studied, standardised prevalence was highest on most measures in participants with schizophrenia and lowest in those with major depression. Mania/BD was the second most impaired exposure group on three of five measures (reasoning, numeric memory and prospective memory), and sensitivity analyses showed that impairment in this group was higher still when exposure information sources were strictly equivalent with the other groups. Reaction time impairment was most prevalent in the MS group, which is in line with previous research showing particular problems with processing speed in MS and other white matter disorders 28, 29, 30. Although the increased prevalence (compared with the unexposed group) of cognitive impairment in major depression was relatively small, lifetime prevalence of major depression is approximately ten times that of BD and schizophrenia and fifty times that of MS and PD, which meant that the population attributable prevalence of cognitive impairment was highest overall for this group. Sensitivity analyses suggested that comorbidities may be contributing to the increased likelihood of cognitive impairment in major depression.

This is the first study to directly compare prevalence of cognitive impairment across these conditions, using consistent assessment methods and a single unexposed comparison group. Multiple sources of information were used to classify exposure status, and impairment status was defined with reference to a very large normative group. Direct standardisation permitted like‐for‐like comparisons across exposure groups, and sensitivity analyses were conducted to examine possible sources of bias and confounding. The overall pattern of findings was consistent regardless of exposure group definitions (wide or narrow) and adjustment for key demographic characteristics.

The prevalence of cognitive impairment in all groups was lower than expected. This may indicate that people living with psychiatric and neurological conditions in the general population are less impaired than the patient population represented in clinical studies. Alternatively, the low prevalence of impairment may reflect selection bias in UK Biobank, such that invitees may have been more motivated to join a medical research study if they had prior experience of health problems, and may have been more willing or able to take part if they had better cognitive function and/or less severe disorder. This would represent collider bias 31, whereby exposed status and (lower) probability of impaired outcome together influenced study participation. This would bias any true association of exposure with impaired outcome towards the null. Similarly, the greater proportion of missing cognitive data in the exposed groups, and the finding that missingness was itself associated with older age, lower educational attainment and comorbidities, suggests that selection into the analysed sample was biased towards more cognitively able participants. It should also be noted that the brevity and limited domain coverage of the cognitive tests may have led to ceiling effects and/or lack of sensitivity to impairment in other aspects of cognitive function, such as long‐term episodic memory and complex executive skills, which are common in psychiatric and neurological conditions.

The prevalence of impairment was higher when mood disorder exposure groups were based on self‐reported doctor diagnosis and/or hospital records, without reference to questionnaire data on lifetime mood disorder experiences (Table S2); the results in Table 2 can therefore be taken as a lower bound of true prevalence. It may be that the questionnaire data misclassified participants as exposed (differentially so for less cognitively impaired participants), or, alternatively, there might be a large number of people living with undiagnosed mood disorders in the general population, whose true prevalence of cognitive impairment is lower than previous studies have suggested.

With regard to demographic factors, our finding that cognitive impairment was less common in women compared with men in the MS group on some measures may reflect generally less severe disease course in women 32; a similar finding for women in the major depression group was unexpected in the light of a recent review, however 33. Impairment was also less common in older compared with younger participants within mania/BD, MS and PD groups. Given the consistent association between older age and greater cognitive impairment in the general population, this finding is likely to indicate survivor bias, whereby only the most able or healthy older individuals in these exposed groups joined UK Biobank or provided complete data for analysis.

A number of study limitations need to be considered. Unlike previous studies in these conditions, clinician‐confirmed diagnoses were not available, and linked health records covered in‐patient and day‐case admissions only. Psychiatric hospital data were also missing for Scotland participants, although these comprised only 7% of the whole cohort. Information regarding exposure status relied substantially on self‐reported diagnoses or responses to questionnaire items. Nevertheless, descriptive data regarding sociodemographic factors, psychological measures and medication use supported the distinctions between the groups. The cross‐sectional nature of the study and the limitations of available clinical information also meant that the onset times and durations of exposures and outcomes were not known; exposure‐outcome associations may not be causal, and it is possible that cognitive impairment may precede clinical onset of some disorders (e.g. schizophrenia). UK Biobank had an invitation response rate of only 6% 22, and the cohort is not representative of the UK population in some respects (e.g. health‐related behaviours such as smoking). The exposure groups that we identified within it are likely to differ from psychiatric and neurological samples in other studies and in clinical practice, with regard to sociodemographic characteristics, illness severity and motivational factors. It is not known whether the degree of non‐representativeness differed across exposure groups, however; if it did not, then between‐group comparisons remain valid. Within the mood disorder groups, it was not possible to distinguish reliably between subtypes of bipolar presentations and single vs. recurrent depression, thus limiting the comparability of the findings to previous clinical studies. Current depressive symptoms were reported, but status with regard to clinical euthymia was not known.

We have previously noted some limitations of the brief cognitive tests 24, 27, and we note here also that the impairment threshold differed slightly across the five tests, because of variation in the reference score distributions. It is possible that the relatively low impairment prevalence on these cognitive tests compared with previous clinical studies reflects insensitivity of the brief measures or differential reliability across exposure groups, but variation in performance across groups was detectable whilst internal consistency was similar. Despite the very large size of the cohort, sample sizes were modest on some cognitive measures and data were sparse across strata in the narrowly defined exposure groups, which limited the standardised analyses. No information was available regarding the impact of cognitive impairment on instrumental functioning: it is possible that the impact of impairment on disability and participation is not well captured by the 5th percentile impairment threshold used here, given that the range of severity below this threshold may differ across disorders, and some people will experience instrumental dysfunction even when measured cognitive performance remains above the 5th percentile threshold.

The present study aimed to quantify prevalence of cognitive impairment in order to permit direct comparisons of burden across disorders of interest. Although we took into account potential confounding influences of age, gender and education, more complex analyses are required to investigate the multitude of other social, clinical, lifestyle and genetic factors that are likely to contribute to cognitive risk and resilience in psychiatric and neurological disorders. It will be possible to explore these in a multivariate explanatory framework using a range of data from baseline and follow‐up assessments in UK Biobank, as well as ongoing extensions to data linkage 34.

## Declarations of interest

I.J.D. is a UK Biobank participant. J.P.P. is a member of the UK Biobank Steering Committee. The authors declare that they have no other competing interests.

## Funding

This research was conducted using the UK Biobank resource, under application 11332. UK Biobank was established by the Wellcome Trust medical charity, Medical Research Council, Department of Health, Scottish Government and Northwest Regional Development Agency. UK Biobank has also had funding from the Welsh Assembly Government and the British Heart Foundation. Data collection was funded by UK Biobank. B.C. is supported by a fellowship from the Scottish Executive Chief Scientist Office (ref. DTF/14/03) and by funding awarded to J.J.E. from The Dr Mortimer and Theresa Sackler Foundation. I.J.D. is supported by The University of Edinburgh Centre for Cognitive Ageing and Cognitive Epidemiology, part of the cross council Lifelong Health and Wellbeing Initiative funded by the Biotechnology and Biological Sciences Research Council (BBSRC) and Medical Research Council (MRC) (MR/K026992/1). D.J.S. is a Lister Institute Prize Fellow. The funders had no role in the study design, analysis or interpretation of data, decision to publish or preparation of the manuscript.

## Supporting information


**Appendix S1** Medications

**Appendix S2** Self‐report diagnoses

**Appendix S3** ICD codes

**Appendix S4** Materials and procedure

**Appendix S5** Sensitivity analyses

**Table S1** Additional Characteristics of the Exposed and Unexposed Groups

**Table S2** Prevalence of Cognitive Impairment Across Groups, Standardised for Age Group, Gender and Educational Attainment

**Table S3** Prevalence of Cognitive Impairment in Alternative Versions of Mood Disorder Groups
