# Supplementary material for: The ‘cognitive footprint’ of psychiatric and neurological conditions: cross‐sectional study in the UK Biobank cohort
Source: Acta Psychiatr Scand. 2017 Apr 7;135(6):593–605. doi: 10.1111/acps.12733 (PMC5434825; doi:10.1111/acps.12733)
Supplement: Supplementary file 4 — Appendix S4 Materials and procedure [file ACPS-135-593-s004.pdf]

SUPPORTING INFORMATION - METHODS

Materials and procedure

Demographic and lifestyle data

Age, gender and ethnic background were self-reported. Neighbourhood-level socio-economic status was measured by the Townsend index of material deprivation (1); this was calculated immediately prior to the baseline assessment, based on the preceding national census output areas. Educational qualifications were self-reported, and for the present study were dichotomised according to whether or not participants held a university/college degree. Self-reported smoking status data were used to classify participants into three categories (current, former and never smokers). Current frequency of alcohol consumption was recorded over six ordinal categories from 'never' to 'daily/almost daily'; participants who responded 'never' were further divided according to whether or not they reported past alcohol consumption ('former drinker'). Current medications were self-reported to the research nurse and were subsequently assigned unique codes. Medication classes of interest were manually coded for the present study: psychotropic medication (lithium, other mood stabilisers, antidepressants, antipsychotics, sedatives or hypnotics); disease-modifying treatments used in MS; and medications used in PD. Appendix 1 lists these medications.

Psychological measures

Measures of neuroticism and current depressive symptoms were administered via touchscreen. Twelve yes/no questions from the Eysenck Personality Questionnaire Revised Neuroticism scale (2) were summed to produce a total score ranging from 0 to 12, with higher scores indicating greater neuroticism. Four questions were administered regarding frequency of depressive symptoms in the past two weeks: depressed mood/hopelessness; unenthusiasm/uninterest; tenseness/restlessness; and tiredness/low energy. Participants self-rated each symptom on a four-point scale from 'not at all' to 'nearly every day', summed to produce an overall score ranging from 0 to 12, with higher scores indicating more frequent depressive symptoms during the preceding two weeks. The neuroticism and current depressive symptom scores were not used in the construction of the exposure groups, and are reported here for descriptive purposes only.

Cognitive function

Brief cognitive tests were administered via touchscreen. The tests were designed specifically for UK Biobank but share some characteristics with other established tests of cognition. Two tests were included in the protocol throughout the UK Biobank baseline phase (reaction time and pairs matching), two tests were introduced in the final two years of recruitment (reasoning and prospective memory), and one test was introduced in the final two years and then subsequently removed for reasons of time (numeric memory). Sample size therefore varies across tests. The total time to complete all five tests was approximately 15 minutes.

<u>Reasoning</u>

Thirteen questions were presented sequentially via touchscreen on a self-paced basis with an overall time limit of two minutes. Responses were selected from a multiple-choice array. Any questions not attempted during the two-minute time limit were scored as zero. The score for analysis was an unweighted total from 0 to 13 (UK Biobank data field 20016, known as the 'fluid intelligence' test), with higher scores indicating better performance.

<u>Reaction time</u>

This test was based on a 'Snap'-style computer game, in which participants were asked to press a button with their dominant hand as quickly as possible each time a matching pair of symbols was

presented on-screen. Five practice trials were administered, followed by seven test trials. The score for analysis was the mean time (in milliseconds) to press the button, derived from the four trials in which a matching pair occurred (UK Biobank data field 20023). Higher scores indicate slower (i.e. worse) performance.

Numeric memory

A string of numbers was presented on-screen, and after a brief delay participants were asked to enter it from memory, in reverse order, via a numeric keypad. Each string was presented on screen for 2000ms, plus an additional 500ms multiplied by the string length. A delay of 3000ms occurred between clearing the screen and activating the response keypad. All participants began with a string length of two, and successive strings increased by one, up to a maximum string length of 12. The test was discontinued after five successive incorrect responses at a string length of two, or after two successive incorrect responses at string lengths of three or more. The score for analysis was the maximum string length recalled correctly (UK Biobank data field 4282), with higher scores indicating better performance.

Pairs matching

Symbol cards were presented on-screen in a random array. Participants were asked to memorise the position of as many matching pairs as possible. The cards were then turned face down on the screen and participants were asked to touch as many matching pairs as possible in the fewest tries. The score for analysis was the number of errors made while attempting to select the pairs, with a higher score indicating worse performance. Two trials of this task were administered, one with three pairs of symbols and one with six pairs. Because there was a ceiling effect on the three-pair trial, only the score on the six-pair trial of the test was analysed in the present study (UK Biobank data field 399.0.2).

Prospective memory

The following instruction appeared on the touchscreen: “At the end of the games we will show you four coloured symbols and ask you to touch the blue square. However, to test your memory, we want you to actually touch the orange circle instead”. After a delay during which participants completed the other cognitive tasks described above, a screen appeared showing four coloured shapes with the instruction to touch the blue square. If the participant touched the orange circle, their response was recorded as ‘correct on first attempt’. If they touched the blue square, they were given a prompt on-screen to try to recall what the original instruction was, and were asked to respond again. If they correctly selected the orange circle after receiving this prompt, their response was recorded as ‘correct on second attempt’. All other responses (including no response) were recorded on the system as incorrect. For the present analyses, data were dichotomised as either ‘correct on first attempt’ or not (derived from UK Biobank data field 20018).

Definition of cognitive impairment

Performance on each of the five tests was classified as impaired or unimpaired. For all measures except prospective memory, the score distribution in the unexposed comparison group was converted into percentile ranks, and the raw score corresponding to the 5th percentile (or 95th, on tests where higher scores represented worse performance) was identified as the cut-off for impairment. If that raw score spanned more than one percentile rank, the nearest percentile rank with a raw score that uniquely divided the sample into impaired and unimpaired groups was used instead. Participants in the exposed groups were then classified as impaired according to that cut-off score, i.e. having a score that was equal to or worse than the lowest-performing 5% (or nearest feasible proportion) of the unexposed group. Since prospective memory was a categorical measure, impairment in all groups was defined as being incorrect on the first attempt.

Minimisation of bias

All assessments were administered according to a standard operating procedure. Administration and scoring of cognitive measures and questionnaires was automated. We aimed to minimise bias in the ascertainment of the exposure groups by using comparable sources of information (self-reported diagnoses and hospital records) for all conditions of interest. Additional questionnaire-based data were, however, only available for the BD and major depression exposures. Participants in Scotland were coded as missing for the mental health exposures, since their hospital records covered general hospital admissions only; any mental health diagnoses in those records would be less likely to be in the primary position compared with participants in England and Wales, therefore possibly reflecting a different clinical presentation or comorbidity status in those participants. A single unexposed comparison group was used as the reference for all exposure groups, so that impairment prevalence ratios could be directly compared.

Data analysis

Statistical analyses were carried out using Stata software version 13 (3). Demographic, lifestyle and psychological measures were summarised descriptively to characterise the exposed and unexposed groups. Townsend deprivation index scores were categorised into quintiles based on frequency in the whole cohort. The reliability (internal consistency) of the cognitive tests in each group was examined using Cronbach's alpha where possible.

Sensitivity analyses

Comorbidity

Because the exposed groups may have had other comorbid psychiatric or brain conditions in addition to the exposure of interest, which might increase cognitive impairment prevalence relative to the unexposed comparison group, sensitivity analyses were conducted to examine the effect on the crude results of restricting the analyses to participants with no known comorbidities.

Educational attainment

Sensitivity analyses were also conducted to examine the effect of accounting for educational attainment in the standardised results. Educational attainment is a potential confounder of cognitive performance that differed across exposure groups in the present study, but including this in the standardisation process may induce bias if it is in fact a consequence of both exposure and cognitive status. Because the typical age of onset of each exposure of interest varies considerably (e.g. in schizophrenia versus PD), it may be that educational attainment is a consequence in exposure groups with younger onset, but a confounder in exposure groups with older onset. Adjusting for a variable that is a consequence of exposure and outcome may cause 'collider bias', inflating the association between exposure and outcome (4). We therefore repeated the age- and gender-stratified standardised analyses with and without additional education stratification, and compared changes in estimates within and between the exposure groups. Further to these analyses, we also checked for an effect of missing data: since there was some missing data on the education variable (but not on age or gender), we compared crude results using all available data versus crude results in only those participants who had complete education data.

Information bias

It is possible that the additional information source (mood history questionnaire) by which participants could be classified as exposed for mood disorders, in the absence of an equivalent information source for the other exposures of interest, might have led to differential

misclassification bias. We therefore constructed alternative versions of the mood disorder exposure groups, only using information from the other two information sources (self-reported diagnoses and linked hospital records), and repeated the analyses for comparison. Additionally, because Scotland hospital records data were included when ascertaining the MS and PD groups but not the psychiatric exposures, the characteristics of the MS and PD groups identified via hospital records were examined with and without Scotland data for comparison. Lastly, because the proportion of missing data on the cognitive measures differed across the exposure groups, the characteristics of those with and without missing data were compared: age, gender, education and comorbidity status were compared within the broadly-defined exposure groups between participants who did and did not have missing data on each cognitive test.

1. TOWNSEND P. Deprivation. J Soc Policy. 1987 Apr;16:125-46.
2. EYSENCK SBG, EYSENCK HJ, BARRETT P. A revised version of the Psychoticism scale. Pers Individ Differ. 1985;6:21-9.
3. STATACORP. Stata Statistical Software: Release 13. College Station, TX: StataCorp LP; 2013.
4. GREENLAND S, PEARL J, ROBINS JM. Causal diagrams for epidemiologic research. Epidemiology. 1999 Jan;10:37-48.
