# Supplementary material for: The ‘cognitive footprint’ of psychiatric and neurological conditions: cross‐sectional study in the UK Biobank cohort
Source: Acta Psychiatr Scand. 2017 Apr 7;135(6):593–605. doi: 10.1111/acps.12733 (PMC5434825; doi:10.1111/acps.12733)
Supplement: Supplementary file 5 — Appendix S5 Sensitivity analyses [file ACPS-135-593-s005.pdf]

SUPPORTING INFORMATION - RESULTS

Sensitivity analyses

Comorbidity
When crude estimates were re-calculated in participants with no known comorbidities, the prevalence ratios attenuated towards the null and some results were no longer statistically significant. In all groups except major depression, estimates generally remained higher compared with the unexposed group, though very small sample sizes in some groups (e.g. schizophrenia and PD) reduced power to detect statistically significant differences or prevented valid analysis. A somewhat different pattern was seen in the major depression group: some estimates in the broadly-defined group reversed such that impairment prevalence became significantly lower compared with the unexposed group, and most estimates in the narrowly-defined group were no longer significantly different from the unexposed group.

Educational attainment
Supplementary Table S2 shows standardised results taking account of educational attainment as well as age group and gender. Results are not reported for some groups owing to sparse data across strata. Compared with results standardised for age and gender only (Table 2), some estimates increased and some decreased in all exposure groups. Magnitude of change in either direction was generally small (the greatest change in prevalence ratio was from 1.92 to 2.22, on the pairs matching test in the narrowly-defined schizophrenia group), and the overall pattern of results remained similar. Educational attainment was lower in the schizophrenia and PD groups compared with the unexposed group, but there was no clear indication of collider bias in the former: inclusion of education in the standardised analyses reduced rather than inflated prevalence estimates on most measures. Comparison of crude results between the full sample and those with complete education data did not indicate an effect of missing data.

Information bias
The analyses were also repeated using alternative versions of the mania/BD and major depression groups, formed without reference to the mood disorders questionnaire data. The results showed that almost all estimates were higher in these alternative groups, and all were significantly higher than in the unexposed group. Crude and standardised results for these groups are presented in Supplementary Table S3.

The characteristics of the MS and PD groups identified via hospital records were very similar regardless of whether Scotland data were included. In participants with an ICD diagnosis of MS, group characteristics including (n = 1,059) and excluding (n = 981) Scotland data were: mean age = 55.3 years (standard deviation [SD] 7.5) and 55.4 (SD 7.4) respectively; women = 72.2% and 72.3%; has a degree = 30.8% and 29.6%; on MS disease-modifying medication = 13.0% and 13.6%; mean reasoning score = 5.7 (SD 2.0) and 5.7 (SD 2.0); median reaction time = 598 (quartile 1 [Q1] 518, Q3 715) and 598 (Q1 520, Q3 719); mean numeric memory score = 6.4 (SD 1.3) and 6.4 (SD 1.3); median pairs matching errors = 3 (Q1 2, Q3 6) and 3 (Q1 2, Q3 6); prospective memory correct = 71.1% and 71.1%. In participants with an ICD diagnosis of PD, group characteristics including (n = 381) and excluding (n = 362) Scotland data were: mean age = 62.3 years (SD 5.6) and 62.2 (SD 5.6) respectively; women = 41.7% and 40.6%; has a degree = 25.5% and 26.6%; on PD medication = 84.9% and 85.0%; mean reasoning score = 5.7 (SD 2.1) and 5.7 (SD 2.1); median reaction time = 578 (Q1 516, Q3 653) and 575 (Q1 512, Q3 653); mean numeric memory score = 6.5 (SD 1.4) and 6.5 (SD 1.4); median pairs matching errors = 4 (Q1 2, Q3 6) and 4 (Q1 2, Q3 6); prospective memory correct = 65.5% and 65.5%.

Comparison of participant characteristics between those with and without missing data on the cognitive measures showed that participants with missing data across each exposed group were generally older, less likely to have a degree, and more likely to have comorbidities. Unexposed comparison participants with missing cognitive data were also older and less likely to have a degree. Men were over-represented in the missing data groups for major depression, schizophrenia and MS, but were under-represented in the missing data group for mania/BD. The missing data mechanism was likely to be 'missing not at random', assuming that participants with worse cognitive function would be more likely to discontinue the tests; impairment prevalence estimates from the complete case analyses reported here are therefore likely to biased downward.
