# Supplementary material for: The ‘cognitive footprint’ of psychiatric and neurological conditions: cross‐sectional study in the UK Biobank cohort
Source: Acta Psychiatr Scand. 2017 Apr 7;135(6):593–605. doi: 10.1111/acps.12733 (PMC5434825; doi:10.1111/acps.12733)
Supplement: Supplementary file 6 — Table S1 Additional Characteristics of the Exposed and Unexposed Groups [file ACPS-135-593-s006.pdf]

Table S1 Additional Characteristics of the Exposed and Unexposed Groups

	Unexposed comparison	Mania/bipolar		Major depression		Schizophrenia		Multiple sclerosis		Parkinson's disease	
		Broad	Narrow	Broad	Narrow	Broad	Narrow	Broad	Narrow	Broad	Narrow
n	104,410	3,020	607	56,425	7,583	850	259	1,905	931	916	323
Ethnic group											
n (%) missing	398 (0.4)	31 (1.0)	4 (0.7)	279 (0.5)	20 (0.3)	21 (2.5)	4 (1.5)	11 (0.6)	8 (0.9)	6 (0.7)	1 (0.3)
White, *n* (%)[a]	94,798 (91.1)	2,727 (91.2)	563 (93.4)	53,515 (95.3)	7,171 (94.8)	719 (86.7)	221 (86.7)	1,859 (98.2)	899 (97.4)	876 (96.3)	310 (96.3)
Asian/Asian British	3,643 (3.5)	84 (2.8)	6 (1.0)	887 (1.6)	144 (1.9)	24 (2.9)	8 (3.1)	5 (0.3)	4 (0.4)	15 (1.7)	6 (1.9)
Black/black British	3,121 (3.0)	84 (2.8)	13 (2.2)	718 (1.3)	108 (1.4)	49 (5.9)	17 (6.7)	11 (0.6)	5 (0.5)	8 (0.9)	1 (0.3)
Other	2,450 (2.4)	94 (3.1)	21 (3.5)	1,026 (1.8)	140 (1.9)	37 (4.5)	9 (3.5)	19 (1.0)	15 (1.6)	11 (1.2)	5 (1.6)
Townsend quintile[b]											
n (%) missing	157 (0.2)	3 (0.1)	1 (0.2)	101 (0.2)	19 (0.3)	3 (0.4)	1 (0.4)	4 (0.2)	2 (0.2)	2 (0.2)	1 (0.3)
Qu1 (least deprived), *n* (%)[a]	18,130 (17.4)	356 (11.8)	71 (11.7)	8,872 (15.8)	1,015 (13.4)	33 (3.9)	8 (3.1)	382 (20.1)	181 (19.5)	222 (24.3)	80 (24.8)
Qu2	21,340 (20.5)	405 (13.4)	78 (12.9)	10,071 (17.9)	1,304 (17.2)	48 (5.7)	11 (4.3)	382 (20.1)	173 (18.6)	188 (20.6)	66 (20.5)
Qu3	21,782 (20.9)	492 (16.3)	99 (16.3)	10,836 (19.2)	1,388 (18.4)	75 (8.9)	19 (7.4)	389 (20.5)	190 (20.5)	177 (19.4)	59 (18.3)
Qu4	23,337 (22.4)	676 (22.4)	133 (22.0)	12,380 (22.0)	1,663 (22.0)	165 (19.5)	44 (17.1)	373 (19.6)	187 (20.1)	167 (18.3)	53 (16.5)
Qu5 (most deprived)	19,664 (18.9)	1,088 (36.1)	225 (37.1)	14,165 (25.2)	2,194 (29.0)	526 (62.1)	176 (68.2)	375 (19.7)	198 (21.3)	160 (17.5)	64 (19.9)
Smoking status											
n (%) missing	372 (0.4)	26 (0.9)	5 (0.8)	236 (0.4)	21 (0.3)	16 (1.9)	4 (1.5)	17 (0.9)	11 (1.2)	10 (1.1)	5 (1.6)
Never, *n* (%)[a]	60,061 (57.7)	1,281 (42.8)	262 (43.5)	27,577 (49.1)	3,611 (47.8)	299 (35.9)	89 (34.9)	897 (47.5)	419 (45.5)	555 (61.3)	201 (63.2)
Former	35,126 (33.8)	1,024 (34.2)	196 (32.6)	20,210 (36.0)	2,559 (33.8)	237 (28.4)	74 (29.0)	690 (36.6)	337 (36.6)	293 (32.3)	98 (30.8)
Current	8,851 (8.5)	689 (23.0)	144 (23.9)	8,402 (15.0)	1,392 (18.4)	298 (35.7)	92 (36.1)	301 (15.9)	164 (17.8)	58 (6.4)	19 (6.0)
Alcohol frequency											
n (%) missing	75 (0.1)	22 (0.7)	4 (0.7)	157 (0.3)	28 (0.4)	13 (1.5)	2 (0.8)	5 (0.3)	4 (0.4)	4 (0.4)	1 (0.3)
Daily/almost daily, *n* (%)[a]	22,026 (21.1)	561 (18.7)	99 (16.4)	10,801 (19.2)	1,361 (18.0)	125 (14.9)	34 (13.2)	368 (19.4)	169 (18.2)	178 (19.5)	62 (19.3)
3-4 times per week	24,920 (23.9)	492 (16.4)	92 (15.3)	11,020 (19.6)	1,281 (17.0)	84 (10.0)	24 (9.3)	342 (18.0)	149 (16.1)	167 (18.3)	50 (15.5)
1-2 times per week	26,928 (25.8)	641 (21.4)	126 (20.9)	13,097 (23.3)	1,634 (21.6)	161 (19.2)	53 (20.6)	447 (23.5)	214 (23.1)	221 (24.2)	75 (23.3)
1-3 times per month	11,266 (10.8)	355 (11.8)	75 (12.4)	7,031 (12.5)	938 (12.4)	79 (9.4)	22 (8.6)	232 (12.2)	120 (12.9)	86 (9.4)	32 (9.9)
Special occasions only	11,449 (11.0)	473 (15.8)	99 (16.4)	8,153 (14.5)	1,207 (16.0)	157 (18.8)	51 (19.8)	295 (15.5)	158 (17.0)	124 (13.6)	55 (17.1)
Never (former drinker)	2,783 (2.7)	296 (9.9)	74 (12.3)	3,609 (6.4)	699 (9.3)	151 (18.0)	46 (17.9)	128 (6.7)	71 (7.7)	79 (8.7)	27 (8.4)
Never (not former drinker)	4,963 (4.8)	180 (6.0)	38 (6.3)	2,557 (4.5)	435 (5.8)	80 (9.6)	27 (10.5)	88 (4.6)	46 (5.0)	57 (6.3)	21 (6.5)
Any psychotropic medication											
n (%) missing	1,180 (1.1)	40 (1.3)	3 (0.5)	949 (1.7)	58 (0.8)	23 (2.7)	7 (2.7)				
n (%)[a]	1,424 (1.4)	1,663 (55.8)	542 (89.7)	23,280 (42.0)	5,530 (73.5)	688 (83.2)	231 (91.7)				
Lithium											
n (%) missing		90 (3.0)	15 (2.5)	1,965 (3.5)	245 (3.2)						
n (%)[a]		501 (17.1)	209 (35.3)	373 (0.7)	117 (1.6)						
Other mood stabiliser											
n (%) missing		75 (2.5)	10 (1.7)	1,930 (3.4)	237 (3.1)						
n (%)[a]		531 (18.0)	267 (44.7)	768 (1.4)	196 (2.7)						
SSRI antidepressant											
n (%) missing		81 (2.7)	15 (2.5)	1,316 (2.3)	123 (1.6)						
n (%)[a]		508 (17.3)	102 (17.2)	15,529 (28.2)	3,792 (50.8)						

	Unexposed comparison	Mania/bipolar		Major depression		Schizophrenia		Multiple sclerosis		Parkinson's disease	
		**Broad**	**Narrow**	**Broad**	**Narrow**	**Broad**	**Narrow**	**Broad**	**Narrow**	**Broad**	**Narrow**
Other antidepressant											
n (%) missing		90 (3.0)	16 (2.6)	1,625 (2.9)	180 (2.4)						
n (%)[a]		428 (14.6)	128 (21.7)	7,239 (13.2)	1,737 (23.5)						
Traditional antipsychotic											
n (%) missing		97 (3.2)	18 (3.0)			57 (6.7)	17 (6.6)				
n (%)[a]		131 (4.5)	39 (6.6)			174 (21.9)	62 (25.6)				
Second generation antipsychotic											
n (%) missing		82 (2.7)	12 (2.0)			43 (5.1)	10 (3.9)				
n (%)[a]		470 (16.0)	205 (34.5)			439 (54.4)	169 (67.9)				
Multiple sclerosis disease-modifying medication											
n (%) missing								153 (8.0)	94 (10.1)		
n (%)[a]								175 (10.0)	120 (14.3)		
Parkinson's disease medication											
n (%) missing										58 (6.3)	11 (3.4)
n (%)[a]										721 (84.0)	300 (96.2)
Neuroticism score											
n (%) missing	17,114 (16.4)	665 (22.0)	130 (21.4)	11,146 (19.8)	1,392 (18.4)	223 (26.2)	67 (25.9)	442 (23.2)	219 (23.5)	200 (21.8)	72 (22.3)
Mean (SD)	3.3 (2.9)	6.8 (3.6)	7.1 (3.6)	6.4 (3.4)	7.4 (3.2)	6.8 (3.6)	6.9 (3.5)	4.6 (3.4)	4.5 (3.3)	4.1 (3.3)	4.4 (3.4)
Current depressive symptom score											
n (%) missing	8,543 (8.2)	311 (10.3)	53 (8.7)	5,585 (9.9)	666 (8.8)	155 (18.2)	45 (17.4)	243 (12.8)	149 (16.0)	113 (12.3)	39 (12.1)
Mean (SD)	1.1 (1.6)	3.6 (3.3)	3.4 (3.2)	3.1 (3.0)	4.0 (3.4)	3.7 (3.3)	3.9 (3.3)	2.6 (2.4)	2.9 (2.5)	2.3 (2.4)	2.6 (2.5)

Abbreviations: Qu, quintile; SD, standard deviation; SSRI, selective serotonin reuptake inhibitor.

[a] Missing excluded from denominator.

[b] Quintiles generated from the whole UK Biobank cohort.
