# Supplementary material for: The ‘cognitive footprint’ of psychiatric and neurological conditions: cross‐sectional study in the UK Biobank cohort
Source: Acta Psychiatr Scand. 2017 Apr 7;135(6):593–605. doi: 10.1111/acps.12733 (PMC5434825; doi:10.1111/acps.12733)
Supplement: Supplementary file 7 — Table S2 Prevalence of Cognitive Impairment Across Groups, Standardised for Age Group, Gender and Educational Attainment [file ACPS-135-593-s007.pdf]

Table S2 Prevalence of Cognitive Impairment Across Groups, Standardised for Age Group, Gender and Educational Attainment

Impairment threshold		Mania/bipolar		Major depression		Schizophrenia		Multiple sclerosis		Parkinson's disease	
		Broad	Narrow	Broad	Narrow	Broad	Narrow	Broad	Narrow	Broad	Narrow
**Reasoning**	*n*	1,866	318	35,211	6,052	274	86	572	272	283	115
≤ unexposed 4th percentile score (Unexposed prevalence 4.16%)	Standardised P %	7.32	7.24	4.33	5.45	10.86	[b]	3.29	[b]	5.95	9.07
	95% CI	6.14, 8.50	4.39, 10.09	4.11, 4.54	4.88, 6.02	7.17, 14.54		1.83, 4.75		3.19, 8.70	3.82, 14.32
	Standardised PR	1.76*	1.74*	1.04[a]	1.31*	2.61*		0.79		1.43	2.18*
	95% CI	1.48, 2.11	1.13, 2.70	0.98, 1.10	1.16, 1.48	1.78, 3.82		0.44, 1.42		0.73, 2.81	1.01, 4.69
**Reaction time**	*n*	2,955	593	55,773	7,484	798	243	1,866	906	897	316
> unexposed 95th percentile score (Unexposed prevalence 4.98%)	Standardised P %	7.22	8.72	5.53	7.02	13.50	11.45	15.34	19.27	6.27	5.98
	95% CI	6.29, 8.15	6.44, 10.99	5.34, 5.72	6.44, 7.60	11.13, 15.87	7.45, 15.46	13.70, 16.97	16.70, 21.84	4.69, 7.86	3.36, 8.59
	Standardised PR	1.45*	1.75*	1.11*[c]	1.41*[d]	2.71*	2.30*	3.08*[e]	3.87*[f]	1.26	1.20
	95% CI	1.26, 1.68	1.30, 2.35	1.06, 1.17	1.28, 1.55	2.21, 3.32	1.52, 3.49	2.70, 3.52	3.30, 4.54	0.92, 1.71	0.73, 1.98
**Numeric memory**	*n*	505	75	10,808	1,746	63	20	193	103	94	35
≤ unexposed 5th percentile score (Unexposed prevalence 5.22%)	Standardised P %	10.07	[b]	5.85	7.88	19.42	[b]	7.20	[b]	[b]	[b]
	95% CI	7.45, 12.70		5.40, 6.29	6.62, 9.15	9.65, 29.19		3.56, 10.85			
	Standardised PR	1.93*		1.12*	1.51*	3.72*		1.38			
	95% CI	1.45, 2.58		1.01, 1.23	1.26, 1.82	2.20, 6.28		0.69, 2.75			
**Pairs matching**	*n*	3,006	606	56,308	7,571	833	256	1,887	920	907	321
> unexposed 95th percentile score (Unexposed prevalence 4.38%)	Standardised P %	6.48	9.15	5.12	5.65	9.68	9.72	6.53	7.97	6.57	7.05
	95% CI	5.60, 7.36	6.86, 11.45	4.94, 5.31	5.13, 6.17	7.67, 11.69	6.09, 13.35	5.41, 7.64	6.22, 9.72	4.96, 8.18	4.25, 9.85
	Standardised PR	1.48*	2.09*	1.17*	1.29*	2.21*	2.22*[g]	1.49*	1.82*	1.50*	1.61
	95% CI	1.28, 1.72	1.59, 2.76	1.12, 1.23	1.16, 1.44	1.74, 2.82	1.38, 3.58	1.21, 1.83	1.39, 2.37	1.14, 1.98	0.98, 2.65
**Prospective memory**	*n*	1,959	335	36,237	6,267	325	103	608	291	306	124
Incorrect score (Unexposed prevalence 22.82%)	Standardised P %	29.89	36.74	22.82	25.56	44.50	42.22	26.24	29.89	27.84	35.60
	95% CI	27.87, 31.92	31.58, 41.90	22.39, 23.25	24.48, 26.64	39.10, 49.90	32.68, 51.76	22.75, 29.74	24.63, 35.15	22.82, 32.86	27.17, 44.03
	Standardised PR	1.31*[h]	1.61*	1.00[i]	1.12*[j]	1.95*	1.85*	1.15	1.31*	1.22	1.56*
	95% CI	1.22, 1.41	1.38, 1.86	0.98, 1.03	1.07, 1.17	1.71, 2.23	1.44, 2.37	0.99, 1.34	1.07, 1.60	0.98, 1.53	1.16, 2.09

Abbreviations: CI, confidence interval; P, prevalence; PR, prevalence ratio.

Standardised estimates are directly standardised by age, gender and education with reference to the unexposed comparison group.

* Significant at $P < 0.05$ (two-tailed).

[a] Significant interaction with gender: women PR = 0.95 (CI 0.88, 1.03); men PR = 1.13 (CI 1.03, 1.24).

[b] Estimates not reported because more than 3 of 8 strata contained no exposed participants with impairment.

[c] Significant interaction with gender: women PR = 1.04 (CI 0.98, 1.10); men PR = 1.21 (CI 1.12, 1.30).

[d] Significant interaction with gender: women PR = 1.28 (CI 1.14, 1.44); men PR = 1.57 (CI 1.34, 1.83).

[e] Significant interaction with age and gender: <60 years PR = 4.29 (CI 3.63, 5.09); ≥60 years PR = 2.43 (CI 1.99, 2.97); women PR = 2.53 (CI 2.20, 2.92); men PR = 3.78 (CI 3.06, 4.66).

[f] Significant interaction with age and gender: <60 years PR = 5.96 (CI 4.91, 7.24); ≥60 years PR = 2.75 (CI 2.12, 3.56); women PR = 2.96 (CI 2.46, 3.57); men PR = 5.01 (CI 3.94, 6.37).

[g] Significant interaction with education: no degree PR = 1.46 (CI 0.76, 2.82); has degree PR = 3.88 (CI 1.96, 7.68).

[h] Significant interaction with age: <60 years PR = 1.41 (CI 1.28, 1.54); ≥60 years PR = 1.24 (CI 1.11, 1.28).

[i] Significant interaction with gender and education: women PR = 0.97 (CI 0.94, 0.99); men PR = 1.04 (CI 1.01, 1.08); no degree PR = 1.03 (CI 0.99, 1.05); has degree = PR 0.94 (CI 0.89, 0.98).

[j] Significant interaction with gender: women PR = 1.06 (CI 0.99, 1.12); men PR = 1.19 (CI 1.10, 1.28).
