# Supplementary material for: The ‘cognitive footprint’ of psychiatric and neurological conditions: cross‐sectional study in the UK Biobank cohort
Source: Acta Psychiatr Scand. 2017 Apr 7;135(6):593–605. doi: 10.1111/acps.12733 (PMC5434825; doi:10.1111/acps.12733)
Supplement: Supplementary file 8 — Table S3 Prevalence of Cognitive Impairment in Alternative Versions of Mood Disorder Groups [file ACPS-135-593-s008.pdf]

Table S3 Prevalence of Cognitive Impairment in Alternative Versions of Mood Disorder Groups[a]

Impairment threshold		Mania/bipolar		Major depression	
		Broad	Narrow	Broad	Narrow
**Reasoning**	*n*	572	152	9,925	816
≤ unexposed 4th percentile score	Crude prevalence %	7.69	10.53	5.95	8.82
(Unexposed prevalence 4.16%)	95% CI	5.51, 9.87	5.65, 15.41	5.48, 6.42	6.87, 10.77
	Crude prevalence ratio	1.85*	2.53*	1.43*	2.12*
	95% CI	1.39, 2.46	1.59, 4.03	1.32, 1.56	1.70, 2.65
	Standardised prevalence %	7.61	11.15	6.07	9.24
	95% CI	5.44, 9.79	6.15, 16.15	5.60, 6.54	7.25, 11.22
	Standardised prevalence ratio	1.83*	2.68*	1.46*[b]	2.22*[c]
	95% CI	1.37, 2.45	1.64, 4.37	1.34, 1.60	1.75, 2.80
**Reaction time**	*n*	1,621	424	30,051	2,123
> unexposed 95th percentile score	Crude prevalence %	8.27	8.25	5.80	7.68
(Unexposed prevalence 4.98%)	95% CI	6.93, 9.61	5.63, 10.87	5.54, 6.06	6.55, 8.81
	Crude prevalence ratio	1.66*	1.66*	1.16*	1.54*
	95% CI	1.41, 1.96	1.21, 2.28	1.10, 1.23	1.33, 1.79
	Standardised prevalence %	8.62	9.51	6.13	8.12
	95% CI	7.25, 9.98	6.72, 12.30	5.85, 6.40	6.96, 9.28
	Standardised prevalence ratio	1.73*	1.91*	1.23*[d]	1.63*
	95% CI	1.46, 2.05	1.37, 2.66	1.17, 1.30	1.40, 1.91
**Numeric memory**	*n*	152	37	2,910	233
≤ unexposed 5th percentile score	Crude prevalence %	12.50	0.00	7.49	11.16
(Unexposed prevalence 5.22%)	95% CI	7.24, 17.76		6.53, 8.45	7.12, 15.20
	Crude prevalence ratio	2.40*		1.44*	2.14*
	95% CI	1.57, 3.66		1.25, 1.65	1.48, 3.08
	Standardised prevalence %	12.95		7.51	11.07
	95% CI	7.61, 18.28		6.56, 8.47	7.04, 15.09
	Standardised prevalence ratio	2.48*		1.44*	2.12*
	95% CI	1.61, 3.81		1.24, 1.66	1.40, 3.21
**Pairs matching**	*n*	1,651	434	30,365	2,151
> unexposed 95th percentile score	Crude prevalence %	7.63	7.37	5.37	6.51
(Unexposed prevalence 4.38%)	95% CI	6.35, 8.91	4.91, 9.83	5.12, 5.62	5.47, 7.55
	Crude prevalence ratio	1.74*	1.68*	1.23*	1.49*
	95% CI	1.47, 2.07	1.20, 2.35	1.16, 1.30	1.26, 1.75
	Standardised prevalence %	7.80	8.23	5.87	7.05
	95% CI	6.50, 9.09	5.65, 10.82	5.60, 6.13	5.97, 8.13
	Standardised prevalence ratio	1.78*[e]	1.88*	1.34*	1.61*
	95% CI	1.50, 2.12	1.33, 2.67	1.26, 1.42	1.36, 1.90
**Prospective memory**	*n*	614	165	10,395	877
Incorrect score	Crude prevalence %	36.64	41.21	26.35	31.58
(Unexposed prevalence 22.82%)	95% CI	32.83, 40.45	33.70, 48.72	25.50, 27.20	28.50, 34.66
	Crude prevalence ratio	1.61*	1.81*	1.15*	1.38*
	95% CI	1.45, 1.78	1.50, 2.17	1.12, 1.19	1.25, 1.53
	Standardised prevalence %	36.74	42.67	27.16	33.09
	95% CI	32.93, 40.55	35.13, 50.22	26.30, 28.01	29.97, 36.20
	Standardised prevalence ratio	1.61*[f]	1.87*	1.19*[g]	1.45*[h]
	95% CI	1.45, 1.79	1.54, 2.26	1.15, 1.23	1.32, 1.61

Abbreviations: CI, confidence interval.
Standardised estimates are directly standardised by age and gender with reference to the unexposed comparison group.
* Significant at $P < 0.05$ (two-tailed).
[a] Exposure groups formed without reference to mood disorder questionnaire data.
[b] Significant interaction with gender: women ratio = 1.31 (CI 1.18, 1.46); men ratio = 1.62 (CI 1.41, 1.87).
[c] Significant interaction with gender: women ratio = 1.65 (CI 1.20, 2.29); men ratio = 2.84 (CI 2.05, 3.92).
[d] Significant interaction with age and gender: <60 years ratio = 1.42 (CI 1.31, 1.54); ≥60 years ratio = 1.13 (CI 1.05, 1.22); women ratio = 1.13 (CI 1.05, 1.21); men ratio = 1.36 (CI 1.25, 1.49).
[e] Significant interaction with age: <60 years ratio = 2.17 (CI 1.74, 2.72); ≥60 years ratio = 1.52 (CI 1.16, 1.98).
[f] Significant interaction with age: <60 years ratio = 1.79 (CI 1.55, 2.07); ≥60 years ratio = 1.46 (CI 1.25, 1.71).
[g] Significant interaction with age and gender: <60 years ratio = 1.25 (CI 1.19, 1.32); ≥60 years ratio = 1.13 (CI 1.08, 1.19); women ratio = 1.12 (CI 1.07, 1.17); men ratio = 1.26 (CI 1.19, 1.34).
[h] Significant interaction with gender: women ratio = 1.31 (CI 1.15, 1.49); men ratio = 1.61 (CI 1.39, 1.88).
